# PBDE Concentrations in Women: Harley et al. Respond

**DOI:** 10.1289/ehp.1002283R

**Published:** 2010-08

**Authors:** Kim G. Harley, Nicholas P. Jewell, Raul Aguilar, Amy R. Marks, Jonathan Chevrier, Asa Bradman, Brenda Eskenazi

**Affiliations:** Center for Children’s Environmental Health Research, School of Public Health, University of California, Berkeley, Berkeley, California, E-mail: kharley@berkeley.edu

In our study (Harley et al. 2010), we found statistically significant associations between higher PBDE (polybrominated diphenyl ether) concentrations in women and longer time to achieve pregnancy. According to Goodman et al., we stated that the association of PBDEs and fecundability is likely causal. We never made this claim. As with all observational studies, associations do not guarantee causation. However, we believe this is a well-conducted study with a strong design to investigate the potential effects of PBDEs on time to pregnancy.

Goodman et al. argue that errors in the measurement of PBDE exposure or in recall of time to pregnancy could bias our results. We agree that little is known about the degree to which PBDE levels vary over time. However, we have no reason to believe that this variability would lead to differential misclassification with regard to the outcome. Similarly, women were blinded as to their PBDE levels, so we have no reason to believe that recall of time to pregnancy was biased. In both cases, measurement error would likely bias our results towards the null, making our results conservative.

Goodman et al. also questioned the appropriateness of our statistical model. The authors correctly point out that a key assumption of the discrete-time Cox proportional hazards model is that the hazard ratio [or, in this case, fecundability odds ratio (fOR)] be constant over the follow-up time. When this assumption is not met, the reported fOR represents a weighted average of the estimate in each month of trying to become pregnant, and may be biased for the exposure effect at a specific time. We tested the proportionality assumption by examining the odds of becoming pregnant in each discrete month when no contraception was used. Although the magnitude of the association was slightly less in the first month of follow-up compared with later months, we found that higher PBDE concentration was associated with decreased fecundability in every month.

Goodman et al. were also concerned about uncontrolled confounding. Although it is true that many factors affect the timing of pregnancy, confounding is present only when these factors are associated with the exposure as well as the outcome. We have no reason to believe that the factors mentioned by Goodman et al. would be associated with PBDE levels, other than by chance. We did evaluate many of the factors they listed (i.e., frequency of intercourse, alcohol consumption, smoking, and drug use) and reported that they did not confound our results. Although we agree that one can never control for all possible confounding factors in an observational study—this is an inherent limitation of epidemiology—we have taken care to minimize confounding as much as possible.

Finally, Goodman et al. argue that limiting the study to pregnant women could bias results away from the null. We cannot think of a circumstance in which this would be true. The inherent selection bias of retrospective studies in pregnant populations is that infertile couples are excluded and subfertile couples are underrepresented. Thus, if there is a true association between PBDEs and time to pregnancy, then limiting the study to the most fertile couples will reduce statistical power and lead to an underestimation of the effect. However, if there is no association between PBDEs and time to pregnancy, then we would expect the fOR to be 1.0 among all women. Overrepresenting the most fertile couples would continue to show a null effect. We fail to see how excluding subfertile women would bias findings away from the null or show a spurious association.

Time-to-pregnancy studies are methodologically complicated, and both prospective and retrospective studies have their limitations. For a detailed discussion of the biases in retrospective study designs, as well as a discussion of how to minimize them, see Joffe et al. (2005). A strength of our study is that we undertook multiple sensitivity analyses to investigate the extent that our findings changed when inclusion criteria or details of the analytic methods were altered. Our findings remained largely unchanged in all these sensitivity analyses. In summary, the limitations pointed out by Goodman et al. would serve to underestimate our estimate of effect, not inflate it. However, since this is the first study of PBDE exposure and time to pregnancy, our findings need to be replicated in other populations.
